# Is the time ripe for new diagnostic criteria of cognitive impairment due to cerebrovascular disease? Consensus report of the International Congress on Vascular Dementia working group

**DOI:** 10.1186/s12916-016-0719-y

**Published:** 2016-11-03

**Authors:** Robert Perneczky, Oren Tene, Johannes Attems, Panteleimon Giannakopoulos, M. Arfan Ikram, Antonio Federico, Marie Sarazin, Lefkos T. Middleton

**Affiliations:** 1 Neuroepidemiology and Ageing Research Unit, School of Public Health, Faculty of Medicine, The Imperial College of Science, Technology and Medicine, Charing Cross Hospital, St Dunstan’s Road, W6 8RP London, UK; 2 Department of Psychiatry and Psychotherapy, Technische Universität München, Ismaninger Str. 22, 81675 München, Germany; 3 Cognitive Impairment and Dementia Service, West London Mental Health NHS Trust, Lakeside Mental Health Unit, West Middlesex University Hospital, Twickenham Road, TW7 6FY London, UK; 4 Department of Psychiatry and Psychotherapy, Ludwig-Maximilians-Universität München, Nussbaumstr. 7, 80336 München, Germany; 5 Department of Psychiatry, Tel Aviv Sourasky Medical Center, Haim Weizman St 6, Tel Aviv-Yafo, 64239 Israel; 6 Sackler Faculty of Medicine, Tel Aviv University, Ramat Aviv, Tel Aviv, 69978 Israel; 7 Institute of Neuroscience, Newcastle University, Campus for Ageing and Vitality, NE4 5PL Newcastle upon Tyne, UK; 8 Department of Psychiatry, University of Geneva School of Medicine, 2 rue Verte, 1205 Geneva, Switzerland; 9 Departments of Epidemiology, Radiology and Neurology, Erasmus MC, ‘s-Gravendijkwal 230, 3015 CE Rotterdam, The Netherlands; 10 Department of Medicine, Surgery and Neuroscience, University of Siena, Viale Bracci 2, 53100 Siena, Italy; 11 Unit of Neurology of Memory and Language, Centre de Psychiatrie et Neurosciences, INSERM UMR S894, Centre Hospitalier Sainte Anne and Université Paris Descartes, Sorbonne Paris Cité, 75013 Paris, France

**Keywords:** Cerebrovascular disease, Dementia, Cognition, Biomarker, Risk factor, Neuroimaging, Cerebrospinal fluid, Magnetic resonance imaging, Genetics, CADASIL

## Abstract

**Background:**

Long before Alzheimer’s disease was established as the leading cause of dementia in old age, cerebrovascular lesions were known to cause cognitive deterioration and associated disability. Since the middle of the last century, different diagnostic concepts for vascular dementia and related syndromes were put forward, yet no widely accepted diagnostic consensus exists to date.

**Discussion:**

Several international efforts, reviewed herein, are ongoing to define cognitive impairment due to cerebrovascular disease in its different stages and subtypes. The role of biomarkers is also being discussed, including cerebrospinal fluid proteins, structural and functional brain imaging, and genetic markers. The influence of risk factors, such as diet, exercise and different comorbidities, is emphasised by population-based research, and lifestyle changes are considered for the treatment and prevention of dementia.

**Conclusion:**

To improve the diagnosis and management of vascular cognitive impairment, further progress has to be made in understanding the relevant pathomechanisms, including shared mechanisms with Alzheimer’s disease; bringing together fragmented research initiatives in coordinated international programs; testing if known risk factors are modifiable in prospective interventional studies; and defining the pre-dementia and pre-clinical stages in line with the concept of mild cognitive impairment due to Alzheimer’s disease.

## Background

The current paper presents the consensus of the Diagnostic Criteria Working Group of the 9th International Congress on Vascular Dementia, held on October 16–18, 2015 in Ljubljana, Slovenia. The concept that late-life dementia can occur in the context of cerebrovascular disease has been known since the nineteenth century [1, 2], and up until the 1960s, cerebral arteriosclerosis as well as widespread white matter lesions in patients with long-standing hypertension were seen as the main cause of dementia in older individuals [1]. This view was challenged by Blessed et al. [3] and others, who suggested that Alzheimer’s disease (AD) neuropathology is the landmark neuropathological feature in the majority of age-related dementia cases. This paradigm shift led to the development of the concept of multi-infarct dementia (e.g. dementia following multiple brain infracts), which subsequently was used to define vascular dementia (VaD) in several international classification systems such as the Diagnostic and Statistical Manual of Mental Disorders 4th edition (DSM-IV) and the International Classification of Diseases and Related Health Problems, 10th edition (ICD-10). However, subsequent studies suggested that the majority of VaD cases were caused by subcortical cerebrovascular changes, rather than by large cortical infarcts [3]. As a result of these conflicting views, different sets of criteria for VaD were developed, including the National Institute of Neurological Disorders and Stroke – Association Internationale pour la Recherche et l’Enseignement en Neurosciences (NINDS-AIREN) [4] and the State of California Alzheimer’s Disease Diagnostic and Treatment Centers criteria [4], as well as criteria for specific subtypes such as subcortical VaD [5]. Parallel to similar developments in the AD field, it was recognised that cerebrovascular disease frequently co-occurs with other pathological changes in the majority of patients over the age of 75 [6], which is the age group when over 70 % of dementia cases occur [7]. Simultaneously, there was controversy regarding the use of the term dementia, which became synonymous with the concept of AD dementia, requiring prominent memory deficits in addition to impairment in at least one other cognitive domain. Nevertheless, this pattern of cognitive decline is not necessarily applicable in patients with VaD [8]. Furthermore, the need for inclusion of pre-dementia changes within a broader nosological concept similar to mild cognitive impairment due to AD was also acknowledged. Based on these considerations, the term ‘vascular cognitive impairment’ (VCI) was proposed [9], which accounts for the heterogeneous nature (and degrees) of cognitive deficits related to prominent cerebrovascular pathologies. Vascular mild cognitive impairment [6] or vascular cognitive impairment, no dementia [7] were terms proposed to categorise the early clinical stages. However, despite VCI being clearly a step in the right direction, it has not been widely adopted and parallel classification systems are still being employed.

Validated clinical diagnostic criteria are important to identify suitable subjects for clinical trials in order to develop new drugs for VCI. Their relevance increases further if treatment strategies are to be developed that target specific pathogenic cerebrovascular mechanisms leading to VCI [10]. Furthermore, lifestyle interventions and other non-pharmacological approaches can only be developed if the target populations are clearly defined [8]. Current efforts towards a consensus on diagnostic criteria and guidelines to account for the heterogeneous nature of VCI, such as the recent International Society for Vascular Behavioural and Cognitive Disorders statement [11], must be encouraged. However, despite considerable recent advances, there are significant gaps in our understanding of the neurobiological mechanisms underpinning the various dementia forms [12]. Further research is therefore required before definitive criteria and guidelines can be formulated. Additionally, the success of such criteria will ultimately depend on a robust pathological and clinical validation and the support of the international research community.

The present paper summarises recent major developments in relation to the clinical diagnosis of VCI. We discuss how this entity is handled in the new DSM-V criteria, how our knowledge about the relevant neuro-pathological changes impacts on the concepts surrounding this diagnosis, and what role fluid and neuroimaging biomarkers play. Further, we briefly summarise the current knowledge about risk factors and how they should be addressed in the context of the clinical diagnostic process. Finally, rare genetic causes of VCI/VaD are discussed and recommendations are provided in relation to the most relevant next steps.

## Vascular cognitive impairment in DSM-V

The fifth edition of DSM, published in 2013 by the American Psychiatric Association, introduced major changes to the chapter referred to in DSM-IV as ‘Dementia, delirium, amnestic, and other cognitive disorders’ [13]. One such change concerns the used nomenclature; the chapter being referred to as ‘Neurocognitive Disorders’ recommends replacing the term dementia with that of ‘major neurocognitive disorder’ (NCD). Two reasons are stated for this change, firstly, dementia is wrongly attributed only to older populations, while it can appear in young adults (e.g. in cases of traumatic brain injury or HIV infection), and, secondly, NCD is a broader definition – individuals with substantial decline in a single domain can receive this diagnosis as opposed to the definition of dementia, which requires a decline in at least two cognitive domains (memory and another domain) [14]. Another substantial change concerns the inclusion of a less severe level of cognitive impairment named mild NCD, which, in DSM-IV, was subsumed under cognitive disorder not otherwise specified. This addition is in line with the current trends in terms of the optimal therapeutic intervention in the very early or pre-symptomatic stages of at-risk individuals for dementia. However, it may also have wide economic and public health implications, adding millions of potential new drug consumers, worldwide [15].

DSM-V also attempts to minimise the use of the ‘not otherwise specified’ category, and stresses the need to identify the presumed underlying cause of the syndrome. Thus, the first step in the diagnostic process is to differentiate between normal neurocognitive function and mild and major NCD, followed by a second step to assign an aetiological category such as Alzheimer’s NCD, vascular NCD or dementia with Lewy bodies (DLB) NCD. In distinguishing among etiological subtypes, additional diagnostic markers are required, such as neuroimaging studies (magnetic resonance imaging (MRI) and positron emission tomography) and other biomarkers. DSM-V evolved from text descriptions of NCD to ‘operationalised’ criteria, so that vascular and other non-AD forms of NCD can now be specifically diagnosed.

Retaining the diagnosis of major or mild vascular NCD implies that cerebrovascular disease is the dominant (if not exclusive) pathology leading to cognitive deficits. DSM-V suggests that such a link is established by determining that either the onset of cognitive deficits is temporally related to one or more cerebrovascular events or that evidence of cognitive decline is prominent in complex attention processes (including processing speed) and frontal-executive function. Furthermore, there should be evidence of the presence of cerebrovascular disease from the history, physical examination and/or neuroimaging to account for the neurocognitive deficits. Finally, it is required that the clinical manifestations are not better explained by any other brain or systemic disorder. In situations where the above criteria are not fully met, DSM-V proposes to use the term of ‘possible’ or ‘probable’ vascular NCD.

Whilst the new multi-dimensional approach, integrating early clinical stages and operationalising aetiological categories, has been well received, the use of positive biomarker data has not been widely adopted in clinical practice. Current diagnostic criteria do not include quantifiable measures for core pathological changes such as degree of small vessel disease (SVD) and many neuroimaging standards for research use have yet to be validated for clinical applications [10]. Furthermore, the lack of a gold standard for measuring processing speed and executive function hampers the standardised assessment of relevant neurocognitive deficits. The diagnosis of VCI in most clinical settings worldwide still relies on traditional descriptive phenomenology (clinical signs and symptoms) and on the exclusion of other possible aetiologies [16]. It is expected that these criteria will be further updated as new knowledge becomes available, both in the areas of neurocognitive testing and biomarkers.

## Neuropathology: vascular dementia versus dementia with mixed pathologies

**Fig. 1:**
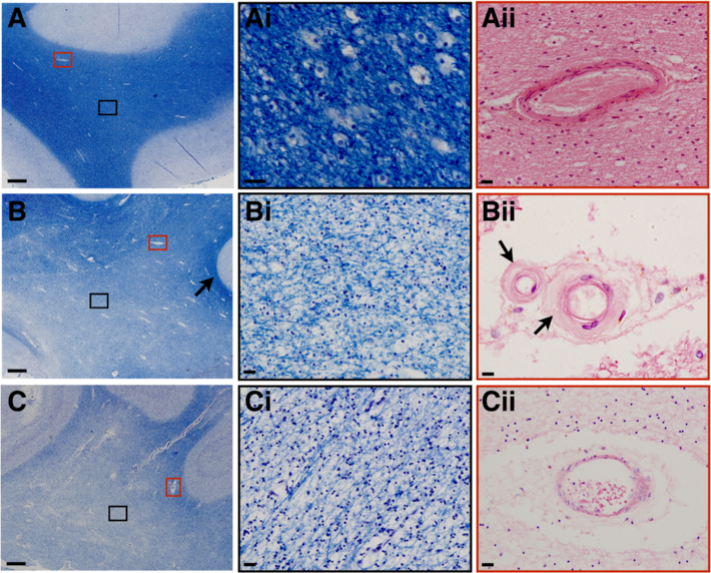
Examples of normal white matter versus severe white matter lesions with and without small vessel disease. Normal white matter and severe white matter lesions of the parietal deep white matter with and without small vessel disease. **A**-**Ai**, normal appearing white matter and a normal white matter artery (**Aii**). **B**, white matter lesion indicated by widespread pallor of the central white matter with typical sparing of the subcortical U-fibres (arrow); **Bi**, higher magnification of white matter lesion exhibiting severe rarefaction, i.e., myelin and axonal loss; **Bii**, white matter arterioles from white matter lesion area exhibing arteriolosclerosis with hyalinisation (arrows) of vessel walls. **C**, white matter lesion with severe white matter pallor; **Ci**, magnifies image of severe white matter rarefaction; **Cii**, white matter arteriole with enlarged perivascular space but no small vessel disease-related fibrosis or hyalinisation. Of note, this case exhibited severe tau pathology in the overlying cortex, suggesting Wallerian-like degeneration to be the cause for white matter damage. Images captured from serial sections. Histological stain Luxol fast blue was used for images **A**, **Ai**, **B**, **Bi**, **C** and **Ci**; H&E stain was used for **Aii**, **Bii** and **Cii**. *Scale bars* represent 1mm in A, B and C and 20μm in **Ai**, **Aii**, **Bi**, **Bii**, **Ci** and **Cii**

It has to be emphasised that the neuropathological diagnosis of VaD remains challenging in the absence of commonly accepted neuropathological criteria.Three main diseases of cerebral blood vessels can contribute to vascular dementia, namely atherosclerosis (AS; large- to medium-sized arteries), SVD (small arteries and arterioles) and cerebral amyloid angiopathy (CAA; arteries, capillaries and rarely veins). AS, SVD and CAA can cause different types of cerebrovascular lesions, including brain infarcts, white matter lesions and cerebral haemorrhages (Fig. 1). Large infarcts can be caused by thrombotic (AS) or thromboembolic (AS, extracranial AS, cardiogenic) occlusion of the vessel’s lumen. Lacunar infarcts are frequently caused by SVD-related vessel occlusion and microinfarcts are often related to SVD (white matter) and CAA (cortex). Based on the pattern of cerebrovascular lesions, three types of dementias associated with cerebrovascular disease may be distinguished, namely multi-infarct dementia, strategic infarct dementia and subcortical vascular encephalopathy (Binswanger’s disease).

In post mortem studies, the prevalence of pure VaD is very low; in a recently reported large series of 6205 participants, only 124 cases (1.9 %) showed cerebrovascular disease as the only morphologic substrate for clinical dementia, compared to > 15 % reported in clinical studies [17]. This discrepancy may be related to the widely held assumption that white matter hyperintensities on MRI are invariably associated with SVD, while recent studies suggest that these are usually associated with cortical neurodegenerative pathology in AD [18] and may also be associated with increased cerebral amyloid load [19]. However, limited cerebrovascular disease is often found in brains of elderly individuals with and without dementia and it is becoming increasingly clear that the ageing brain is characterised by the presence of multiple pathologies rather than the characteristic neuropathological features of one single neurodegenerative disease only. For instance, limited Lewy body pathology is seen in over 40 % of AD patients and DLB virtually always shows limited AD pathology [20], whilst a TDP-43 pathology has been demonstrated in over 50 % of AD cases, having a rather distinct topographical spreading pattern affecting the clinical presentation [20]. Nevertheless, the examples above should not be interpreted as ‘mixed dementia’ cases, where the presence of two distinct neurodegenerative diseases fulfilling all neuropathological criteria for each disease (e.g. AD and DLB) must be present [21]. Therefore, AD associated to limited cerebrovascular pathology should not be referred to as mixed dementia. However, we should be aware that even in cases that both clinically and neuropathologically fulfil the criteria for a single dementing disease, the presence of additional pathologies is likely. While the extent and severity of such additional neuropathological lesions is not sufficient to cause dementia, they may influence clinical symptoms and contribute to the pathogenesis of dementia, but their respective impact remains to be elucidated.

## Risk factors and epidemiology

An important cornerstone in understanding dementia and in developing preventive and interventional strategies is to first unravel the risk factors of disease, especially those that are potentially modifiable. Therefore, herein, we will consider lifestyle and environmental factors and not dwell into genetic risk factors. Interestingly, although AD and VaD, as well as their pre-dementia counterparts mild cognitive impairment due to AD and vascular mild cognitive impairment, are still considered distinct entities, their risk factor profiles overlap substantially. Several large population-based cohort studies (such as the Framingham Heart Study, Rotterdam Study and the Cardiovascular Health Study) have shown that established vascular factors (such as hypertension, diabetes mellitus, smoking and hyperlipidaemias) are risk factors of dementia, including AD as well as VaD [11]. Additionally, a range of clinical cardiac diseases are also independent risk factors of dementia, including atrial fibrillation [12], myocardial infarction [13] and heart failure [15]. More recently, markers of subclinical cardiac dysfunction have been linked to clinical dementia as well as preclinical brain damage identified on MRI [16].

Taken together, this evidence has formed the basis of two important research questions that have shaped epidemiological research on dementia in recent years. First, what proportion of the total burden of dementia is, in fact, caused by vascular risk factors? Second, if vascular factors are important in dementia, including AD, has improved cardiovascular health in recent decades had an effect on dementia occurrence?

Several studies have now shown that up to a third of all dementia cases can be attributed to cardiovascular risk factors [17–19]. This proportion is not restricted to VaD cases, but in fact includes all dementia cases, the majority of which may be due to AD. Importantly, this indicates that, if cardiovascular risk factors could entirely be removed from a population, theoretically, a third of all dementia cases would be preventable. This then leads to the second question of whether improvement of cardiovascular risk management has had an effect on dementia occurrence over the last few decades. Already in 2012, the Rotterdam Study presented evidence suggesting that the incidence of dementia was lower in the period 2000–2005 compared to 1990–1995 [21]; this finding was subsequently corroborated in several other Western studies [22–24] and the decrease seems to be attributable to better cardiovascular prevention. Indeed, a recent lifestyle intervention randomised controlled trial (RCT) provided, for the first time, direct evidence of a putative preventive effect of better cardiovascular control on cognitive decline [25]. If these results are further supported by a number of on-going non-pharmacological lifestyle intervention RCTs, there will be important worldwide public health implications in formulating effective preventive strategies in the wider population.

## Cerebrospinal fluid (CSF) markers

Biomarkers can be measured in a wide range of body fluids, including blood, urine and saliva, but the CSF has been studied most extensively in relation to cerebral nervous system disorders because it often reflects biochemical processes in the brain more accurately compared to markers in the periphery. Nevertheless, CSF biomarker studies have been scarce for VCI, compared to AD. Some biomarker candidates, such as matrix metalloproteinases [26], have been proposed, but the evidence thus far remains unconvincing.

Because of the significant overlap between AD and cerebrovascular pathology, the established AD protein markers total-tau, phosphorylated-tau and amyloid-beta (Aβ)_42_ may be promising candidates for VCI/VaD. In a recently reported evaluation of over 5000 patients with dementia in Sweden [27], VaD and AD were statistically assigned to two independent clusters when a combination of all three AD markers was used for the classification. Approximately 15 % of VaD and 60 % of mixed VaD cases had AD-typical CSF biomarker profiles. Interestingly, over 50 % of VaD cases had low Aβ_42_ concentrations, which may suggest the presence of concomitant Aβ pathology. The CSF measurements in this study were performed in clinical practice and might therefore have influenced the clinical diagnostic process, introducing a risk of circular reasoning and highlighting the need of prospective biomarker studies in VCI. Overall, these findings support the notion that a combined analysis of the three established AD CSF markers may be most useful in the differential diagnosis of VaD and VCI in more general terms.

In addition to the search for reliable cerebrovascular biomarkers, the contribution of cerebrovascular changes to the pathophysiology of AD and other late-onset dementias are increasingly being recognised [28]. Therefore, the development and validation of vascular biomarkers may, indeed, have diagnostic applications in AD and other dementia types, in addition to VCI. This notion is highlighted by the two-hit vascular hypothesis of AD [29], which proposes that microvascular damage may be the initial insult that leads to the dysfunction of the blood–brain barrier and/or decreased brain perfusion resulting in secondary neuronal injury and paving the way for the accumulation of neurotoxic Aβ oligomers. A leakage of neurotoxic proteins into the brain results from blood–brain barrier disruption, followed by a response from microglia and astrocytes, angiogenesis, and neuro-inflammation. These processes can promote white-matter damage, formation of toxic tau neurofibrillary tangles, loss of dendritic spines and Aβ accumulation. Despite the suspected linkage between vascular and AD pathology, the contribution of vascular changes to AD pathophysiology has not been adequately addressed in most studies of AD pathophysiology. The majority of biomarker studies are narrow in scope, investigate only a single category of brain injury or exclude certain patient groups, e.g. those with significant vascular risk factors or damage. In order to successfully develop new improved biomarkers for VCI and AD, future studies will need to be more comprehensive, both in terms of the targeted biomarkers and patient populations.

## Neuroimaging of vascular changes

Structural neuroimaging techniques are of pivotal importance for the differential diagnosis of VaD, but also for elucidating the pathophysiological mechanisms surrounding the development of small vascular lesions in brain aging. In routine clinical settings, computed tomography (CT) and MRI provide evidence of vascular lesions that are rarely specific of a given diagnostic entity with the marked exception of cerebral autosomal dominant arteriopathy with subcortical infarcts and leucoencephalopathy (CADASIL). In CADASIL, there are subcortical white matter lesions affecting the temporal pole and hyperintensity signals on T2-weighted images mainly in pons in the absence of low intensity T1 signals in deep brain stem and cerebellum [30].

**Fig. 2:**
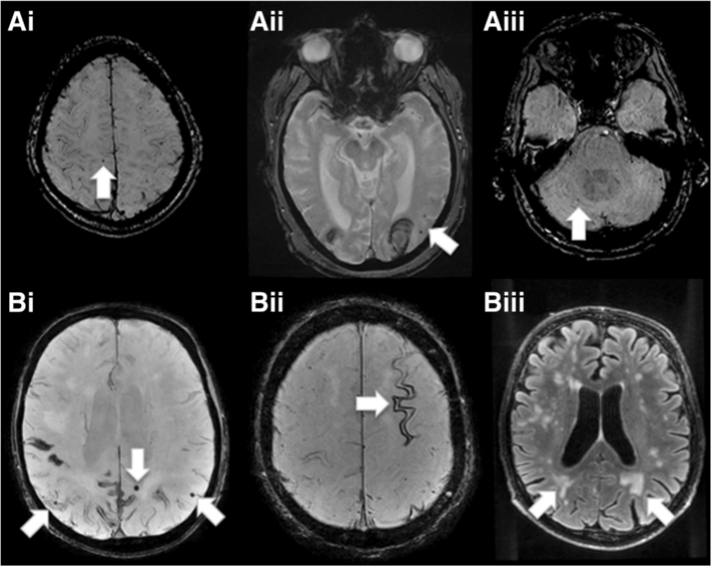
Examples of typical brain magnetic resonance imaging changes associated with vascular cognitive impairment. *A Hypertensive cerebral microbleeds*. Typical appearance of hypertensive cerebral microbleeds in patients around 70 years of age. Note the random distribution including supratentorial superficial white matter (**Ai**), intraparenchymal (**Aii**), and infratentorial region (**Aiii**). *B Cerebral amyliod angiopathy*. Typical manifestaion of celebral amyloid angiopathy in a 72 years old patient, including multiple microbleeds with a labor distribution sparing the deep grey matter and infratentorial region (**Bi**), superficial siderosis of the covexity (**Bii**) and periventricular leucoencephalopathy (**Biii**)

**Fig. 3:**
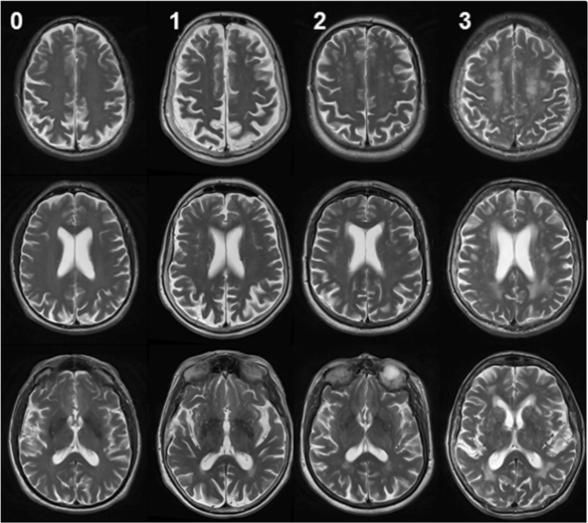
Examples of typical magnetic resonance imaging of white matter disease. *White matter lesions*. Usually attributed to chronic small vessel ischaemia, rating using the Fazekas scale. No lesions or single punctate lesion (grade 0), multiple punctate lesions (grade 1), beginning confluency of lesions (bridging, grade 2), large confluent lesions (grade 3)

Besides these rare cases, it is well established that most clinically overt VCI cases evolve progressively over several years via the accumulation of small vascular and microvascular lesions, mainly in subcortical regions. Subsequently, the construct of VCI has been proposed to capture the entire spectrum of cognitive disorders associated with all forms of cerebral vascular brain injury, not solely stroke, ranging from mild cognitive impairment through to fully developed dementia. As already proposed by the NINDS-AIREN criteria and subsequently confirmed by neuroimaging studies, a substantial percentage of VaD cases is due to SVD affecting small cerebral arterioles, capillaries and venules possibly due to intrinsic arteriolar occlusive disorder [31]. Although the small vessels are not easily identifiable, detailed neuroimaging may allow for tracking of their pathology in the human brain. The main imaging features of SVD visible on 1.5 T and 3 T MRI scans are acute lacunar infarcts, neurologically silent lacunes, white matter hyperintensities, increased perivascular spaces and microbleeds [32, 33] (Figs. 2 and 3). Microinfarcts are strongly related to cognitive decline, yet they are detectable only at higher field strengths, usually only available in research facilities [34]. Lacunar strokes correspond to lesions less than 20 mm in axial diameter; old lacunes are small cavities between 3 and 15 mm in diameter located in the deep grey or white matter [27]. Both these lesions are known to affect cognition, mainly in pure VaD cases [35]. On MRI scans, they are detected by increased signal on diffusion-weighted imaging, reduced signal on an apparent diffusion coefficient map, increased signal on fluid-attenuated inversion recovery, increased T2-weighted imaging, reduced signal on T1-weighted MRI, and low attenuation on CT scanning. White matter hyperintensities are areas of decreased attenuation in CT scans, increased signal on T2 and fluid-attenuated inversion recovery sequences, and in some cases decreased on T1-weighted MRI scans. Present in periventricular and deep white matter, in the basal ganglia (deep grey matter) and more rarely in pons and cerebellum, they signal the progression of SVD and their severity is related to the emergence of VaD in clinical samples [36]. Increased perivascular spaces are visible on T2- and T1-weighted MRI scans mostly in basal ganglia and subcortical white matter. Strongly related to white matter hyperintensities, they are very frequent in old age but the increase of their number is related to VCI [37]. Finally, cortical microbleeds are punctiform areas of hypointensity on T2- or susceptibility-weighted sequences, of up to 1 cm of diameter. An impressive number of studies have attempted to define their cognitive significance with conflicting results [38–40]; their negative impact on cognition is highly likely in VaD, but much less in mixed cases.

In addition to microvascular and small macrovascular lesions, recent contributions pointed to the presence of white matter microstructure changes assessed with diffusion tensor imaging (DTI) at the early stages of cognitive decline (reviewed in [41]). Several DTI-derived parameters were taken into account, including fractional anisotropy and longitudinal, radial and mean diffusivity. Tract-based spatial statistics were used to project DTI-derived data onto a mean tract skeleton by using non-linear registration. The tract skeleton is the basis for voxel-wise cross-subject statistics and reduces potential misregistrations as the source for false-positive or false-negative analysis results. Applying voxel-wise DTI analyses, a significant decrease of fractional anisotropy values was detected in hemispheric deep white matter and corpus callosum in patients with VCI compared to healthy controls [42]. Although still in its infancy, the automatic processing of DTI data at an individual level using support vector machine analysis may allow identification of the very first signs of biological compromise of white matter in VCI.

## Familial forms of vascular dementia

Herein, we briefly summarise the available data in relation to clinical diagnostic criteria. The genetic underpinning of cerebrovascular changes has been less well studied compared to other dementia forms, such as AD [43]. However, in most genetic studies in sporadic AD, the inclusion criteria are such that they may allow a variable number of cases and/or of mixed pathologies to be included. Of note, the upper age limit of most of these studies is in excess of 80 years, when mixed pathologies are common. Thus far, studies have failed to characterise the heritability of sporadic VCI but there have been several reports on rare monogenic conditions involving cerebral small vessels and predisposing to ischemic and/or haemorrhagic stroke and diffuse white matter disease. In these disorders, the primary genetic defect (autosomal dominant, recessive or X linked) may alter endothelial cells of microvessels leading to impairment of deep brain vascularisation and resulting in clinical manifestations such as subcortical leucoencephalopathy and episodes of stroke.

A diagnosis of hereditary cerebral small vessel disease has to be considered in familial cerebrovascular disorders occurring mainly in young adulthood; it can be distinguished from sporadic cerebral microangiopathy due to the presence of a high vascular risk load. The most important clinical entities are CADASIL, cerebral autosomal recessive arteriopathy with subcortical infarcts and leucoencephalopathy, COL4A1-related cerebral small vessel diseases, autosomal dominant retinal vasculopathy with cerebral leuco-dystrophy, and Fabry disease. The main clinical and genetic characteristics of these disorders are presented in Table 1. Although they have variable phenotypes and different defective genes, all these diseases cause arteriopathy and microvascular disintegration leading to VCI. In these cases, genetic, biochemical or pathological analyses will confirm the clinical suspicion and are very useful in clinical practice (Table 2) [4, 44, 45]. Specific substitution treatment with recombinant enzymes is available only for Fabry’s disease, and treatment mainly improves non-central nervous system organ function (kidney, heart, peripheral nerve, etc.), with little change in central neurological abnormalities because of the difficulty of the enzyme to cross the blood–brain barrier [46].

**Table 1:** Characteristics of important inherited cerebral small vessel diseases

Disease	Gene	Protein	Onset age	Clinical features
CADASIL	NOTCH3 (autosomal dominant)	Notch3 receptor protein	30–40 years	Progressive dementia, mood disorders, migraine, recurrent subcortical cerebral, infarction On MRI, leucoencephalopathy, mainly in temporal poles
CARASIL	HTRA1 (autosomal recessive)	HTRA1, serine protease	20–30 years	Mood changes, pseudobulbar palsy, mental dysfunction, scalp alopecia in the teen, acute mid-to-lower back pain
				Subcortical white matter changes on MRI
				Heterozygous autosomal dominant form: later age of onset and absence of typical extraneurological features
COL4A1	COL4A1 (autosomal dominant)	Type IV collagen α1-chain	All ages	Ischemic stroke, intracerebral haemorrhage, retinal arteriolar tortuosity, cataracts, glaucoma, anterior segment dysgenesis of the eye (Axenfeld–Rieger anomaly), muscle cramps, Raynaud phenomena, kidney defects
RVCL	TREX1 (autosomal dominant)	Trex1 DNAse III	30–40 years	Retinal vasculopathy, TIA, strokes, cognitive dysfunction, headaches, personality disorders, Raynaud’s phenomena, liver and kidney dysfunction
Fabry disease	alpha-GalA (X-linked)	Alpha-galactosidase (α-GalA)	Childhood	Classic form: acroparesthesias, angiokeratomas, hypohidrosis, characteristic corneal and lenticular opacities, proteinuria, peripheral neuropathy, TIA and stroke, heart disturbances and cardiomyopathy Heterozygous females: milder symptoms, later onset

*CADASIL* cerebral autosomal dominant arteriopathy with subcortical infarcts and leucoencephalopathy, *CARASIL* cerebral autosomal recessive arteriopathy with subcortical infarcts and leucoencephalopathy, *COL4A1* COL4A1-associated diseases, *RVCL* retinal vasculopathy with cerebral leucodystrophy, *TIA* transient ischemic attack

**Table 2:** In vivo diagnosis of genetic small vessel diseases

Disease	Genetic investigations	Pathological investigations	Biochemical investigations
CADASIL	NOTCH3 mutations	Evidence of granular osmiophilic material in affected arterioles	None
CARASIL	HTRA1 mutations	N/A	N/A
COL4A1	COL4A1 type IV collagen α1-chain	N/A	N/A
RVCL	TREX1 DNAse III	N/A	N/A
Fabry disease	Alpha Gal-A gene mutations	Lysosomal abnormalities in tissues	Deficiency a-galactosidase activity in serum, urine, leucocytes, tissues; abnormalities in urinary and tissues glycolipids

*CADASIL* cerebral autosomal dominant arteriopathy with subcortical infarcts and leucoencephalopathy, *CARASIL* cerebral autosomal recessive arteriopathy with subcortical infarcts and leucoencephalopathy, *COL4A1* COL4A1-associated diseases, *RVCL* retinal vasculopathy with cerebral leucodystrophy

## Recommendations and future perspectives

Despite significant progress in recent years, important gaps in knowledge persist in terms of the extent and granularity of dementia types and their distinct underlying biological pathways, as well as the mechanisms underpinning cognitive deterioration. There is abundant evidence that mixed pathologies are very prevalent in patients over the age of 75 and that pure VCI is rare. It is also evident that cognitive profiles may differ between different types of dementia and that the AD-centred definition of dementia may not be appropriate for other dementia types. We have also learned, from large-scale clinical trials, that AD-specific treatments, such as cholinesterase inhibitors and memantine, may not show the desired effects in people with other dementias. Imaging and fluid biomarkers are useful in differentiating AD from VCI and to enrich trial populations with relatively pure cases. However, significant pathological (and presumably nosological) overlaps do exist between the diagnostic groups, even in biomarker-based clinically diagnosed cases, whereas biomarkers are still not part of the diagnostic armamentarium in the majority of clinical dementia settings worldwide.

In view of these significant gaps of knowledge, further research is urgently required to address key issues. Firstly, our understanding of the key pathogenic mechanisms of VCI is far less developed than for AD. They include not only the determinants of vascular burden in the human brain but also its indirect consequences (increased neuro-inflammation, altered oxidative stress regulation, changes in brain reactivity) over time. Further, shared patho-mechanisms between VCI and AD remain poorly understood in view of their complexity. Secondly, large population-based studies have identified important risk factors for late-life dementia and some are, indeed, modifiable, thus promising targets for prevention and treatment strategies. This important hypothesis now needs to be scrutinised in appropriately powered prospective pharmacological and non-pharmacological RCTs. Thirdly, fragmented research initiatives must be brought together in coordinated international research programs in order to optimally utilise human, financial and other resources and increase power. For example, large-scale prospective longitudinal studies aimed at identifying genetic and other risk factors may also identify predictive and diagnostic markers. The accumulated information may well allow for a better delineation and definition of dementia types, such as AD and VCI and, potentially, their subtypes. We have learned from other disease areas, such as cancer, that this is a pre-condition for the discovery and development of effective new medicines. Finally, the pre-dementia and pre-clinical stages of VCI have to be defined more precisely and we propose that the term vascular mild cognitive impairment is used in line with the concept of mild cognitive impairment due to AD to characterise the early clinical stage that precedes VaD.

## Abbreviations

AD: Alzheimer’s disease
AS: Atherosclerosis
Aβ: Amyloid-beta
CAA: Cerebral amyloid angiopathy
CADASIL: Cerebral autosomal dominant arteriopathy with subcortical infarcts and leucoencephalopathy
CSF: Cerebrospinal fluid
CT: Computed tomography
DLB: Dementia with Lewy bodies
DSM: Diagnostic and Statistical Manual of Mental Disorders
DTI: Diffusion tensor imaging
ICD-10: International Classification of Diseases and Related Health Problems, 10th edition
MRI: Magnetic resonance imaging
NCD: Neurocognitive disorder
NINDS-AIREN: National Institute of Neurological Disorders and Stroke – Association Internationale pour la Recherche et l’Enseignement en Neurosciences
RCT: Randomised controlled trial
SVD: Small vessel disease
VaD: Vascular dementia
VCI: Vascular cognitive impairment
